# Mild autonomous cortisol secretion and brain function: cognitive, structural, and functional correlates

**DOI:** 10.3389/fendo.2026.1913085

**Published:** 2026-08-20

**Authors:** Laura Ziccardi, Andrea Romano, Giulia Moltoni, Antonella De Carolis, Maria Elena Aloini, Michele Cavallari, Guido Roberto, Alessandro Bozzao, Franco Giubilei, Antonio Stigliano

**Affiliations:** 1 Sant’Andrea Hospital, Department of Neuroscience, Mental Health and Sensory Organs (NESMOS), Sapienza University of Rome, Rome, Italy; 2 Endocrinology, Department of Clinical and Molecular Medicine, Sant’Andrea Hospital, Faculty of Medicine and Psychology, Sapienza University of Rome, Rome, Italy; 3 Centre for Neurological Imaging, Brigham and Women’s Hospital, Harvard Medical School, Somerville, MA, United States

**Keywords:** adrenal adenoma, adrenal tumor, cognitive function, hippocampal connectivity, mild autonomous cortisol secretion (MACS), resting-state fMRI, sleep quality

## Abstract

**Context:**

Mild autonomous cortisol secretion (MACS) is increasingly recognized as a condition associated with subtle cognitive, functional, and clinical alterations, despite the absence of overt hypercortisolism. Its impact on brain function, sleep quality, and mood, however, remains incompletely understood.

**Objective:**

The aim of this observational, cross-sectional study was to investigate cognitive performance, sleep quality, mood, and structural and functional brain alterations in patients with MACS compared with those with non-functioning adrenal tumors (NFAT).

**Participants:**

Forty-eight patients with adrenal incidentalomas were enrolled, including 28 patients with MACS and 20 with NFAT, all without clinical signs of overt hypercortisolism at study entry.

**Methods:**

Participants had a confirmed failure to suppress cortisol on 1 mg of dexamethasone and underwent comprehensive neuropsychological testing, sleep quality assessment (PSQI), mood evaluation (BDI-II), and brain magnetic resonance imaging (MRI) including structural imaging, voxel-based morphometry, and resting-state functional MRI (fMRI).

**Results:**

Compared with patients with NFAT, those with MACS exhibited slightly lower Mini-Mental State Examination (MMSE) scores and poorer delayed verbal recall, both negatively correlated with serum cortisol levels. Patients with MACS also showed significantly poorer sleep quality, with MACS status and female sex emerging as independent predictors. No group differences were observed in depressive symptoms. Resting-state fMRI revealed altered hippocampal connectivity in MACS, with reduced connectivity with the caudate nucleus and increased connectivity with mesial occipital regions. Voxel-based morphometry showed no structural differences between groups.

**Conclusions:**

Even mild, clinically silent cortisol excess can be associated with subtle cognitive alterations, impaired sleep, and early functional connectivity changes, despite preserved brain morphology. Longitudinal studies are needed to determine whether these alterations progress over time and whether they improve with targeted treatment.

## 1 Introduction

Adrenal nodules are frequently detected in clinical practice, often incidentally during imaging for unrelated reasons (1). Their prevalence ranges from 3% to 10%, increasing with age and showing a slight female predominance (2). Most of these lesions involve the adrenal cortex and are non-functioning adrenal tumors (NFAT). However, approximately half of them are characterized by mild autonomous cortisol secretion (MACS) (3). Currently, MACS is defined in patients who lack clinical signs of overt Cushing’s syndrome but have post-dexamethasone 1 mg (DST-1mg) serum cortisol concentrations above 50 nmol/L (>1.8 µg/dL) (1). Although often clinically silent, MACS is associated with increased morbidity and mortality (4–7). Cortisol plays a crucial role in brain function, modulating stress adaptation via mineralocorticoid receptors (MRs) and glucocorticoid receptors (GRs) (8). MRs are abundant in the cingulate cortex, amygdala, inferior frontal cortex, and cerebral vessels, whereas GRs predominate in the occipital and parietal lobes (8). Physiologically, cortisol secretion is regulated by a biological clock within the hypothalamic–pituitary–adrenal (HPA) axis. Chronic cortisol exposure induces HPA axis dysregulation, which is implicated in several neurological disorders, including depressive disorders (9) and cognitive impairments, such as deficits in attention and memory (10). Furthermore, cortisol excess has been recognized as a cause of impaired myelination, reduced white matter integrity, and inhibition of oligodendrocyte proliferation, leading to axonal demyelination. These effects may promote cortisol resistance, neuroinflammation, and small vessel damage, contributing to blood–brain barrier dysfunction via activation of microglia and astrocytes (8). Morphological studies in Cushing’s syndrome have revealed global brain atrophy, with significant impairment in structures involved in emotional and cognitive functions, such as the hippocampus, anterior cingulate cortex, middle frontal gyrus, and amygdala (11, 12).

Clinically, these alterations are associated with a broad spectrum of disorders, including memory loss, deficits in verbal learning (10), impaired concentration, increased anxiety (13), psychosis, and apathy, potentially progressing to cognitive decline and dementia (14). Unfortunately, in many cases, neuropsychological traits may persist even after hypercortisolism remission, suggesting possible irreversible damage (15).

Increasing data support the idea that MACS reflects a biological continuum in hormone secretion, associated with different degrees of cortisol excess (1, 16). These findings suggest that prolonged exposure to mild cortisol over time may negatively affect brain function. Indeed, elevated plasma cortisol has been associated with faster cognitive decline and poorer temporal-lobe-related functions. While overall cognitive performance may appear preserved, impairments in memory, attention, visuospatial abilities, and executive functions have been observed (17). Cortisol levels have been found to correlate with performance in these domains. Recent evidence also documents neuropsychiatric alterations in patients with MACS, including increased sleep disturbances, heightened perceived stress, and lower perceived social support, often in the absence of formally diagnosed psychiatric disorders. Moreover, adrenal adenomas are associated with an increased risk of depression and anxiety (18–21).

Despite increasing evidence on the impact of cortisol excess, most studies have focused on structural brain magnetic resonance imaging (MRI), with limited exploration of functional alterations. Functional MRI (fMRI), however, can detect early disruptions that may precede structural damage and cognitive decline. Moreover, neuropsychological symptoms, sleep disturbances, and mood alterations are increasingly recognized even in subclinical forms of hypercortisolism but remain underexplored in a systematic and integrative way. This study aims to fill these gaps by combining structural and functional MRI with comprehensive neuropsychological, sleep, and mood assessments in patients with MACS and those with NFAT.

## 2 Materials and methods

### 2.1 Study design, participant recruitment, and endocrine assessment

This observational, cross-sectional study was conducted between March 2022 and January 2025 at Sant’Andrea Hospital in Rome, Italy. The study was carried out in accordance with the principles of the Declaration of Helsinki, adopted by the World Medical Association (WMA) at its 18th General Assembly in Helsinki, Finland. Ethical approval was obtained from the local ethics committee (Prot n. 0020/2026), and written informed consent was obtained from all participants after they received detailed information about the study.

Participants were recruited during follow-up visits at the Adrenal Pathology Unit. All patients were subjected to a hormonal biochemical study at baseline where they were dosed with glucocorticoids, mineralocorticoids, androgen, metanephrine, and cortisol after 1-mg overnight dexamethasone suppression tests (DST-1 mg) according to the European Society of Endocrinology and the European Network for the Study of Adrenal Tumors (ESE/ENSAT) current criteria practice guideline (1). Patients aged 50–80 years with a diagnosis of NFAT or MACS were included. Diagnosis was based on repeated 1-mg overnight dexamethasone suppression tests (DST-1 mg), performed during at least three different visits to confirm persistent failure of cortisol suppression. Dexamethasone was administered the night before cortisol measurement, which was obtained the following morning at 8:00 a.m. after at least 8 h of fasting. For each participant, the mean of the three post-DST-1-mg cortisol measurements was used for analysis. Patients with mean cortisol ≥50 nmol/L, in the absence of clinical signs or symptoms of hypercortisolism, were classified as MACS according to ESE/ENSAT criteria; in these patients, all three post DST-1mg cortisol values were consistently ≥50 nmol/L. Patients with post-DST-1-mg cortisol concentrations <50 nmol/L in all assessments were classified as NFAT.

Patients were excluded if they met any of the following criteria: medications interfering with dexamethasone metabolism, incorrect dexamethasone intake, failure to adhere to the protocol, estrogen-containing therapy [interfering with corticosteroid-binding globulin (CBG) levels], diabetes, hepatic or renal impairment, psychiatric disorders, current use of psychotropic medications, major neurological disease, inability to complete neuropsychological testing, significant hearing or visual impairment, comorbidities affecting cognitive function, substance or alcohol abuse, history of head trauma, fewer than 5 years of education, and body mass index (BMI) > 35. Furthermore, only patients with adequately controlled hypertension and dyslipidemia were included. Former smokers were included only if smoking cessation had occurred more than 10 years before enrollment.

Plasma ACTH levels were measured to confirm ACTH-independent cortisol secretion. All patients included had baseline ACTH values no higher than 21.2 pg/mL (median ± IQR: 10 ± 11.8). ACTH measured in parallel with cortisol on DST-1mg was <5 pg/mL in order to exclude patients with ACTH-dependent hypercortisolism (median ± IQR: 3 ± 2). Cortisol was assayed using a chemiluminescent microparticle immunoassay (Alinity, Abbott, Chicago, IL, USA), while plasma ACTH levels were measured using a chemiluminescent immunoassay (Liaison XL, DiaSorin, Italy). All hormonal measurements were performed in the same certified laboratory of Sant’Andrea Hospital using standardized procedures throughout the study period to minimize inter-assay variability. All participants had previously undergone adrenal computed tomography (CT) or MRI, demonstrating an adrenal adenoma larger than 10 mm. Imaging study was performed using a protocol including a preliminary non-enhanced phase and a contrast-enhanced study based on an arterial, venous, and delayed phase. The images from the CT scanner were evaluated according to a quantitative analysis: (i) baseline Hounsfield Unit (HU) measurement; (ii) calculation of the absolute and relative wash-out according to the formulas: RPW (absolute washout percentage) = HU after 60 s from contrast medium − HU after 15 min/HU after 60 s × 100; APW (percentage of relative wash-out) = HU after 60 s from contrast medium − HU after 15 min/HU after 60 s – HU without contrast medium × 100. Adrenal lesions with HU < 10 were considered benign and excluded pheochromocytoma.

Multi-planar (axial, sagittal, and coronal) T1/T2 fast MRI included a slice thickness 3 to 4 mm max and an interslice gap interleaved or minimal gap (0.3 mm or 10% of slice thickness) to prevent missing small nodules. The protocol included T2-weighted TSE/FSE, T2-weighted TSE with Fat Sat, T1-weighted Dual-Echo GRE, DWI (Diffusion Weighted Imaging), T1-weighted 3D GRE, and Dynamic Contrast-Enhanced (DCE) imaging. The protocol included sequence captured with T1-weighted 3D fat-suppressed gradient echo, followed by contrast medium (0.1 mmol/kg). The absolute washout (AW) calculation was performed according to the formula: SI venous − SI delayed/SI venous − SI pre-contrast × 100. Adrenal lesions markedly reducing signal intensity when switching from in-phase to out-of-phase images, containing intracellular fat, were considered adenoma.

Overall, 48 participants fulfilled all eligibility criteria and completed the study procedures. Of these, 20 were classified as NFAT and 28 were categorized as MACS.

### 2.2 Neuropsychological assessment

All participants underwent a comprehensive neuropsychological assessment administered by the same trained examiner to ensure consistency across evaluations; the examiner was blinded to participants’ endocrine status. Global cognitive functioning was assessed using the Mini-Mental State Examination (MMSE) (22). A broad range of cognitive domains was evaluated using standardized neuropsychological tests validated in the Italian population and supported by normative data. The cognitive domains assessed are detailed below:

- General Intelligence/Logical Reasoning: Raven’s Colored Progressive Matrices (RCPM)—total number of correct responses (23)
- Processing Speed: Trail Making Test—Part A (24) (TMT-A)—time to completion (seconds); Semantic Fluency (25)—total number of words generated within 60 s in response to semantic cues.
- Selective Attention: Visual Search Test (25)—total number of correctly identified targets within 45 s across three trials.
- Short-Term Memory: Verbal: Digit Span Forward (26)—longest correctly repeated sequence of orally presented digits. Visual: Corsi Block-Tapping Test (26)—longest correctly reproduced sequence of blocks previously indicated by the examiner.
- Long-Term Memory (Episodic Memory): Verbal: Rey Auditory Verbal Learning Test (RAVLT) (23)—number of words recalled after a 15-min delay following five learning trials. Visual: Rey–Osterrieth Complex Figure Test (ROCF) (23)—recall of the figure after a 15-min delay.
- Constructional Praxis/Visuospatial Abilities: ROCF (23)—Copy Condition—number of correctly reproduced elements of the figure.
- Semantic-Lexical Processing: Semantic Fluency (25)—number of words generated in 60 s based on semantic categories; Phonemic Fluency (25)—number of words generated in 60 s based on phonological cues.
- Executive Functions: Trail Making Test (24)—Parts A and B—difference in time between Part B and Part A (B–A); Phonemic Fluency (25) as described above.

### 2.3 Sleep quality evaluation

Sleep quality was assessed using the Pittsburgh Sleep Quality Index (PSQI), in accordance with previous literature (27, 28). The questionnaire was administered at the same time as the neuropsychological assessment. The PSQI is a self-report instrument that evaluates sleep quality over a 1-month period. It provides a global score and seven component scores, taking into account several sleep variables, including subjective sleep quality, sleep latency, sleep duration, habitual sleep efficiency, sleep disturbances, use of sleep medications, and daytime dysfunction. Each component is scored from 0 to 3, yielding a global score ranging from 0 to 21, with higher scores indicating poorer sleep quality. A global PSQI score >5 has been validated as highly sensitive and specific for distinguishing poor from good sleepers (29). Both the dichotomous classification (poor vs. good sleepers) and the continuous global score were used in the analyses.

### 2.4 Depressive symptoms evaluation

The presence and severity of depressive symptoms were assessed using the Beck Depression Inventory, Second Edition (BDI-II), a widely used and well-validated self-report instrument (30, 31). It comprises 21 items that evaluate cognitive, affective, somatic, and vegetative symptoms of depression, referring to the individual’s experiences over the preceding 2 weeks. Each item is rated on a four-point scale ranging from 0 to 3, yielding a total score between 0 and 63. Total scores are interpreted using standard thresholds: 14–19 indicates mild depression, 20–28 denotes moderate to severe depression, and scores above 28 suggest severe depression (32).

### 2.5 Imaging protocol

All patients underwent a brain MRI examination at a 1.5-T scanner (GE Signa Voyager) with a 32-channel phased-array head coil. Besides the conventional morphological sequences, fMRI protocol included the following:

1. The resting-state fMRI obtained with a gradient-echo echo-planar imaging (EPI) sequence (TR 3,000 ms, TE 35 ms, flip angle 90°, FOV 240 × 240 mm, matrix 64 × 64, slice thickness 4 mm without gap, 36 slices, 128 volumes, bandwidth = 250 Hz/pixel). During the resting-state fMRI acquisition, subjects have been instructed to stay awake, relax with their eyes closed, and remain motionless as much as possible. Each scan session lasted 6 min.
2. The anatomical images: a sagittal three-dimensional T1-WI sequence employing a magnetization prepared rapid gradient echo (MPRAGE) over the whole brain.
3. All scans were inspected by an expert neuroradiologist to ensure the absence of MRI artifacts or excessive movement before analysis.

#### 2.5.1 Functional imaging processing

Results included in this manuscript come from analyses performed using CONN (RRID: SCR_009550) release 22.a (33) and SPM (34) (RRID: SCR_007037) release 12.7771.

#### 2.5.2 Preprocessing

Functional and anatomical data were preprocessed using a flexible preprocessing pipeline (35) including removal of initial scans, realignment with correction of susceptibility distortion interactions, slice timing correction, outlier detection, direct segmentation and MNI-space normalization, and smoothing. The first five scans in each functional run were removed. Functional data were realigned using SPM realign & unwarp procedure (36), where all scans were co-registered to a reference image (first scan of the first session) using a least squares approach and a six-parameter (rigid body) transformation (37) and resampled using b-spline interpolation to correct for motion and magnetic susceptibility interactions. Temporal misalignment between different slices of the functional data was corrected following SPM slice-timing correction (STC) procedure (38, 39) using sinc temporal interpolation to resample each slice BOLD time series to a common mid-acquisition time. Potential outlier scans were identified using AR (40) as acquisitions with framewise displacement above 0.5 mm or global BOLD signal changes above three standard deviations (41), and a reference BOLD image was computed for each subject by averaging all scans excluding outliers. Functional and anatomical data were normalized into standard MNI space, segmented into gray matter, white matter, and cerebrospinal fluid (CSF) tissue classes, and resampled to 2-mm isotropic voxels following a direct normalization procedure (42) using SPM unified segmentation and normalization algorithm (43, 44) with the default IXI-549 tissue probability map template. Last, functional data were smoothed using spatial convolution with a Gaussian kernel of 10-mm full width at half maximum (FWHM).

#### 2.5.3 Denoising

Functional data were denoised using a standard denoising pipeline (45) including the regression of potential confounding effects characterized by white matter time series (five CompCor noise components), CSF time series (five CompCor noise components), motion parameters and their first-order derivatives (12 factors) (46), outlier scans (below 99 factors) (41), session effects and their first-order derivatives (2 factors), and linear trends (2 factors) within each functional run, followed by bandpass frequency filtering of the BOLD time series (47) between 0.008 and 0.09 Hz. CompCor (48, 49) noise components within white matter and CSF were estimated by computing the average BOLD signal as well as the largest principal components orthogonal to the BOLD average, motion parameters, and outlier scans within each subject’s eroded segmentation masks. All subjects were included in the analysis, with the denoising procedure optimized to retain the maximum number of effective degrees of freedom (41).

#### 2.5.4 Voxel-based morphometry

T1-weighted MR images of the brain were preprocessed using the voxel-based morphometry (VBM) tool of the SPM12 software package (Wellcome Department of Imaging Neuroscience, London, UK) based on MATLAB R2023a (The MathWorks, Natick, MA, USA). First, the structural images were segmented into GM, WM, and CSF images by using a unified segmentation procedure after image-intensity nonuniformity correction was performed. The segmented GM images were then nonlinearly transformed using diffeomorphic anatomical registration (DARTEL) techniques. Subsequently, the images were modulated to create a modified template for DARTEL based on the MNI152 template provided by SPM12, followed by smoothing with an 8-mm FWHM isotropic Gaussian kernel.

### 2.6 Statistical analysis

#### 2.6.1 Neuropsychological evaluation, sleep, and depression assessment

Analyses were conducted on the final sample of 48 participants (20 NFAT and 28 MACS). Statistical analyses of cognitive performance, sleep quality, and depression symptoms were conducted using IBM SPSS Statistics, version 26.0. Descriptive statistics summarized demographic and clinical characteristics of the study population. The normality of variables was assessed using the Shapiro–Wilk test and data transformations were applied when necessary.

Continuous variables are presented as mean ± standard deviation (SD) if normally distributed, or as median and interquartile range (IQR) if non-normally distributed. Categorical variables are reported as absolute frequencies and percentages.

Group comparisons were performed using appropriate statistical tests based on the data distribution including independent samples *t*-tests, Mann–Whitney *U* tests and one-way analysis of variance (ANOVA). The chi-square test was used for comparisons of categorical variables.

The strength and direction of associations between variables were assessed using Pearson’s and Spearman’s correlation coefficients, depending on the data distribution. Partial correlations controlling for potential confounders (e.g., age and education) were performed where appropriate.

Binary logistic regression analyses were performed to investigate whether endocrine status (MACS vs. NFAT) was independently associated with poor sleep quality and depressive symptoms after adjustment for potential confounders, including age, sex, and education. Covariates included in multivariable models were selected *a priori* based on clinical relevance and to avoid model overfitting. To further evaluate whether endocrine status was independently associated with cognitive performance, generalized linear models (GLMs) were fitted for selected cognitive outcomes identified in the primary between-group analyses. Models included MACS status as the main predictor and were adjusted for age, sex, and years of education. Additional models were further adjusted for PSQI score to assess whether sleep quality influenced the observed associations. Regression coefficients (*B*), 95% confidence intervals (95% CI), and corresponding *p*-values are reported. A two-tailed *p*-value < 0.05 was considered statistically significant.

#### 2.6.2 Image analysis—functional imaging

First-level analysis SBC_01: Seed-based connectivity maps (SBC) and ROI-to-ROI connectivity matrices (RRC) were estimated, characterizing the patterns of functional connectivity with 164 HPC-ICA networks (33) and Harvard–Oxford atlas ROIs (50). Functional connectivity strength was represented by Fisher-transformed bivariate correlation coefficients from a weighted GLM (51), defined separately for each pair of seed and target areas, modeling the association between their BOLD signal time series. In order to compensate for possible transient magnetization effects at the beginning of each run, individual scans were weighted by a step function convolved with an SPM canonical hemodynamic response function and rectified.

Group-level analyses were performed using a GLM (51). For each individual voxel, a separate GLM was estimated, with first-level connectivity measures at this voxel as dependent variables (one independent sample per subject and one measurement per task or experimental condition, if applicable), and groups or other subject-level identifiers as independent variables. Voxel-level hypotheses were evaluated using multivariate parametric statistics with random-effects across subjects and sample covariance estimation across multiple measurements. Inferences were performed at the level of individual clusters (groups of contiguous voxels). Cluster-level inferences were based on parametric statistics from Gaussian Random Field theory (52, 53). Results were thresholded using a combination of a cluster-forming *p* < 0.001 voxel-level threshold, and a familywise corrected *p*-FDR < 0.05 cluster-size threshold (54, 55).

#### 2.6.3 Image analysis—voxel-based morphometry

The obtained GM images were analyzed using the SPM12 statistical software package to compare the differences between groups. NFA and MACS groups were compared using two-sample *t*-test. Potential confounding variables, such as age and sex, were included as covariates in the model.

## 3 Results

### 3.1 Study participants

Forty-eight individuals (mean age ± SD: 62.52 ± 6 years) with adrenal incidentaloma were enrolled, including 20 patients with NFAT and 28 with MACS. The characteristics of the study population are summarized in **Table 1**.

**Table 1:** Characteristics of the study participants.

Variable	Total (*N* = 48)	NFAT (*N* = 20)	MACS (*N* = 28)	*p*-value
Age (mean ± SD)	62.52 ± 6	61.05 ± 4.96	64.18 ± 6.62	0.08
Sex, *N* (%) Female Male	28 (58%) 20 (42%)	12 (60%) 8 (40%)	16 (57%) 12 (43%)	0.84
Years of education (median ± IQR)	13 ± 5	13 ± 5	13 ± 6	0.92
BMI (median ± IQR)	27.35 ± 6.87	27.8 ± 6.43	36.65 ± 5.40	0.196
Post-1-mgDST cortisol (nmol/L) (median ± IQR)	75.43 ± 57.13	33.84 ± 8.6	105.13 ± 58.6	**<0.01**
Baseline ACTH (median ± IQR)	10.00 ± 11.80	14 ± 13.28	8.5 ± 7.65	**0.041**
Post-1-mgDST ACTH (median ± IQR)	3.00 ± 2.00	2.85 ± 2.32	3.5 ± 1.35	0.093
Hypertension, *N* (%)	21 (44%)	7 (35%)	14 (50%)	0.30
Dislipidemia, *N* (%)	13 (27%)	7 (35%)	6 (21%)	0.30

NFAT, non-functional adrenal tumors; MACS, mild autonomous cortisol secretion; 1-mg-DTS, dexamethasone suppression test 1 mg.

Bold values are significant results, specifically with a p value < 0.05.

Patients with NFAT were slightly younger than those with MACS; however, no statistically significant differences were observed between groups regarding sex distribution, education level, or comorbidities. As expected, patients with MACS showed significantly higher post-1-mg DST cortisol levels than those with NFAT (105.13 ± 58.6 vs. 33.84 ± 8.6 nmol/L, *p* < 0.001; Mann–Whitney *U* test).

### 3.2 Cognitive performance

The cognitive data are shown in **Table 2**. Overall, neuropsychological performance was within the normal range for age and education in both groups according to the available Italian normative data; however, the NFAT group showed a slightly better cognitive performance than MACS, as measured by the MMSE score adjusted for age and education (mean ± SD: 29.45 ± 1.23 vs. 28.46 ± 1.92; *p* = 0.036, *t*-test). Conversely, the MACS group showed better phonemic fluency (adjusted for age and education) than the NFAT group (mean ± SD: 37.57 ± 8.54 vs. 32.42 ± 7.84; *p* = 0.043, *t*-test). A trend toward better performance on the Trail Making Test B was observed in the MACS group, although the difference did not reach statistical significance (mean ± SD: 46.11 ± 33.72 vs. 70.95 ± 60.48; *p* = 0.076, *t*-test). We found no other differences between the two groups in cognitive performance. We also analyzed the relationship between serum cortisol levels and cognitive performance in the whole sample of 48 study participants. Partial correlation analyses adjusted for age and education revealed a significant negative correlation between post-1-mg DST cortisol levels and MMSE scores (*r* = −0.396, *p* = 0.010), as well as delayed recall on the RAVLT (*r* = −0.320, *p* = 0.041). Serum cortisol levels were inversely associated with immediate recall on the RAVLT, although the association did not reach statistical significance (*r* = −0.263; *p* = 0.097). In GLMs using MMSE as the dependent variable, MACS status was independently associated with lower MMSE scores after adjustment for age, sex, and education (*B* = −0.426, 95% CI −0.830 to −0.022, *p* = 0.039). This association remained significant after further adjustment for PSQI score (*B* = −0.442, 95% CI −0.872 to −0.013, *p* = 0.044), suggesting that the association between MACS and global cognitive performance is independent of sleep quality. Similarly, in GLMs using phonemic fluency as the dependent variable, MACS status showed a positive association with phonemic fluency after adjustment for age, sex, and education (*B* = 4.795, 95% CI −0.035 to 9.626, *p* = 0.052). After additional adjustment for PSQI score, the association was further attenuated and did not reach statistical significance (*B* = 4.322, 95% CI −0.607 to 9.251, *p* = 0.086).

**Table 2:** Neuropsychological test scores adjusted for age and education in the NFAT and MACS groups, with corresponding *p*-values.

Variable (mean ± SD)	NFAT (*N* = 20)	MACS (*N* = 28)	*p*-value
MMSE	29.5 ± 1.2	28.5 ± 1.9	**0.036**
Visual search test	48.7 ± 6.5	49.6 ± 7.6	0.664
Trail making test (a)	32.3 ± 15.9	26.4 ± 14.2	0.186
Trail making test (b)	71.0 ± 60.5	46.1 ± 33.7	0.076
Trail making test (b-a)	40.8 ± 48	22.6 ± 20.9	0.081
Digit span forward	6.2 ± 1	6 ± 1.2	0.601
Corsi block-tapping test	5.1 ± 0.6	4.8 ± 0.7	0.230
Rey Auditory Verbal Learning Test	7.9 ± 2.9	7.7 ± 2.9	0.793
Rey–Osterrieth Complex Figure Test (immediate recall)	15.5 ± 5.1	13.8 ± 6.4	0.324
Rey–Osterrieth Complex Figure Test (delayed recall)	15.2 ± 4.6	14.5 ± 5.9	0.650
Phonemic fluency	32.4 ± 7.8	37.6 ± 8.5	**0.043**
Semantic fluency	42.1 ± 6.7	44 ± 10.4	0.471
Rey–Osterrieth Complex Figure Test (copy)	32.8 ± 6.5	33.6 ± 4.6	0.604
Raven’s Colored Progressive Matrices	30.9 ± 4.7	30.3 ± 4.5	0.672

NFAT, non-functional adrenal tumors; MACS, mild autonomous cortisol secretion.

Bold values are significant results, specifically with a p value < 0.05.

### 3.3 Sleep quality

**Figure 1:**
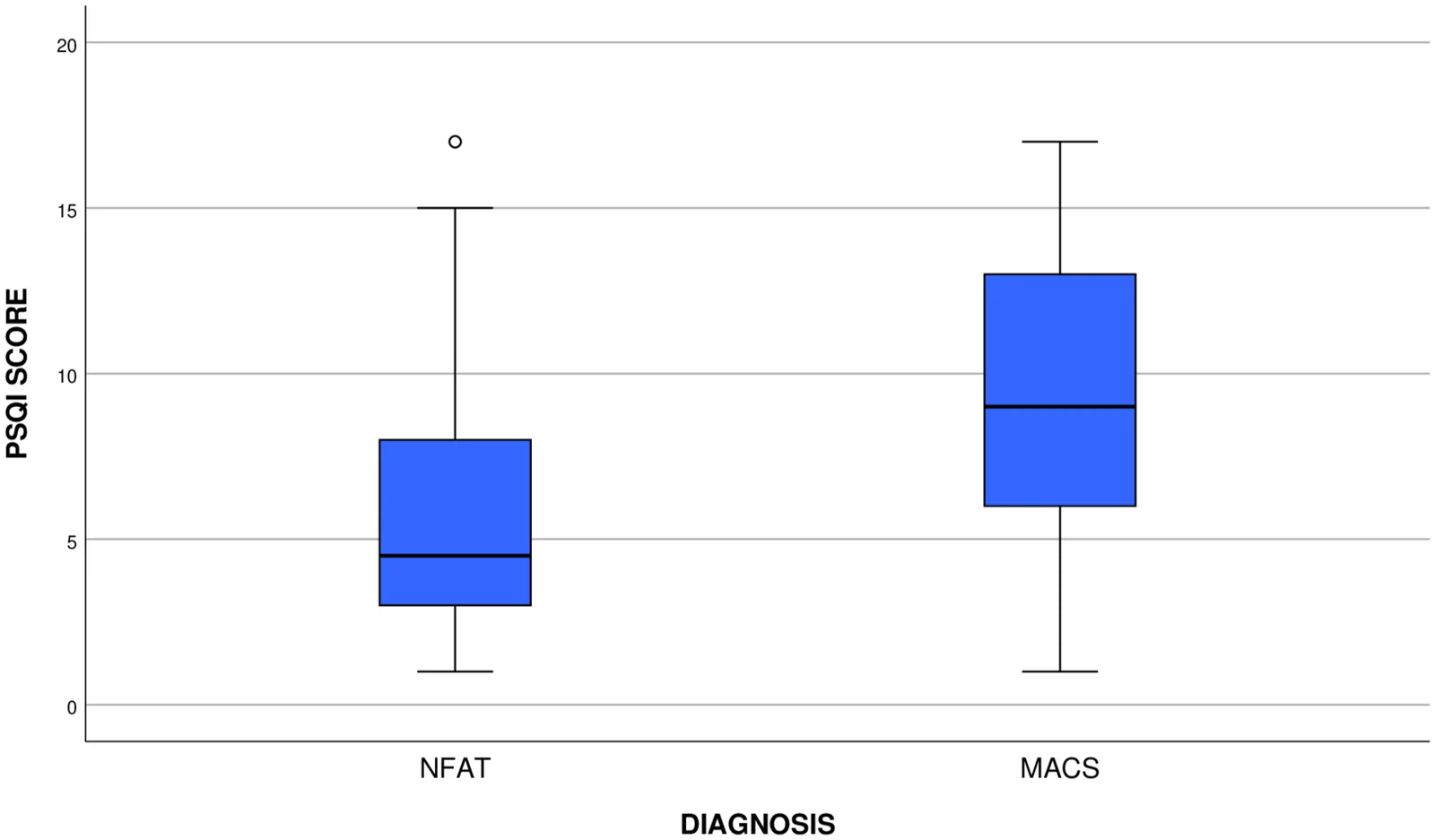
Differences in PSQI scores between the NFAT and MACS groups, box plot. The box represents the interquartile range (IQR) with the median indicated by the central line; whiskers extend to 1.5 × IQR. Circles represent the outliers. NFAT, non-functional adrenal tumors; MACS, mild autonomous cortisol secretion; PSQI, Pittsburgh Sleep Quality Index.

Patients with MACS exhibited poorer sleep quality than those with NFAT, both when sleep quality was analyzed as a dichotomous variable (*p* = 0.017, chi-square test) and when considering PSQI global scores (*p* = 0.040, Mann–Whitney *U* test, **Figure 1**). Post-1-mg DST cortisol levels were positively correlated with PSQI scores, although the association did not reach statistical significance (Spearman’s ρ = 0.236, *p* = 0.107).

Binary logistic regression analysis adjusted for age, sex, and education revealed that MACS status and female sex, but not age, were independently associated with poor sleep quality (MACS: OR = 6.1, CI: 1.217 to 30.520, *p* = 0.028, female sex: OR = 7.6, CI: 1.654 to 35.267, *p* = 0.009).

To explore whether sleep quality contributed to the observed cognitive differences, associations between PSQI and MMSE scores were examined. No significant correlation was found between PSQI and MMSE scores (Spearman’s ρ = −0.117, *p* = 0.427). Likewise, in GLMs with MMSE as the dependent variable, PSQI score was not independently associated with MMSE performance (*B* = −0.053, 95% CI −0.153 to 0.046, *p* = 0.292).

### 3.4 Depressive symptoms

**Figure 2:**
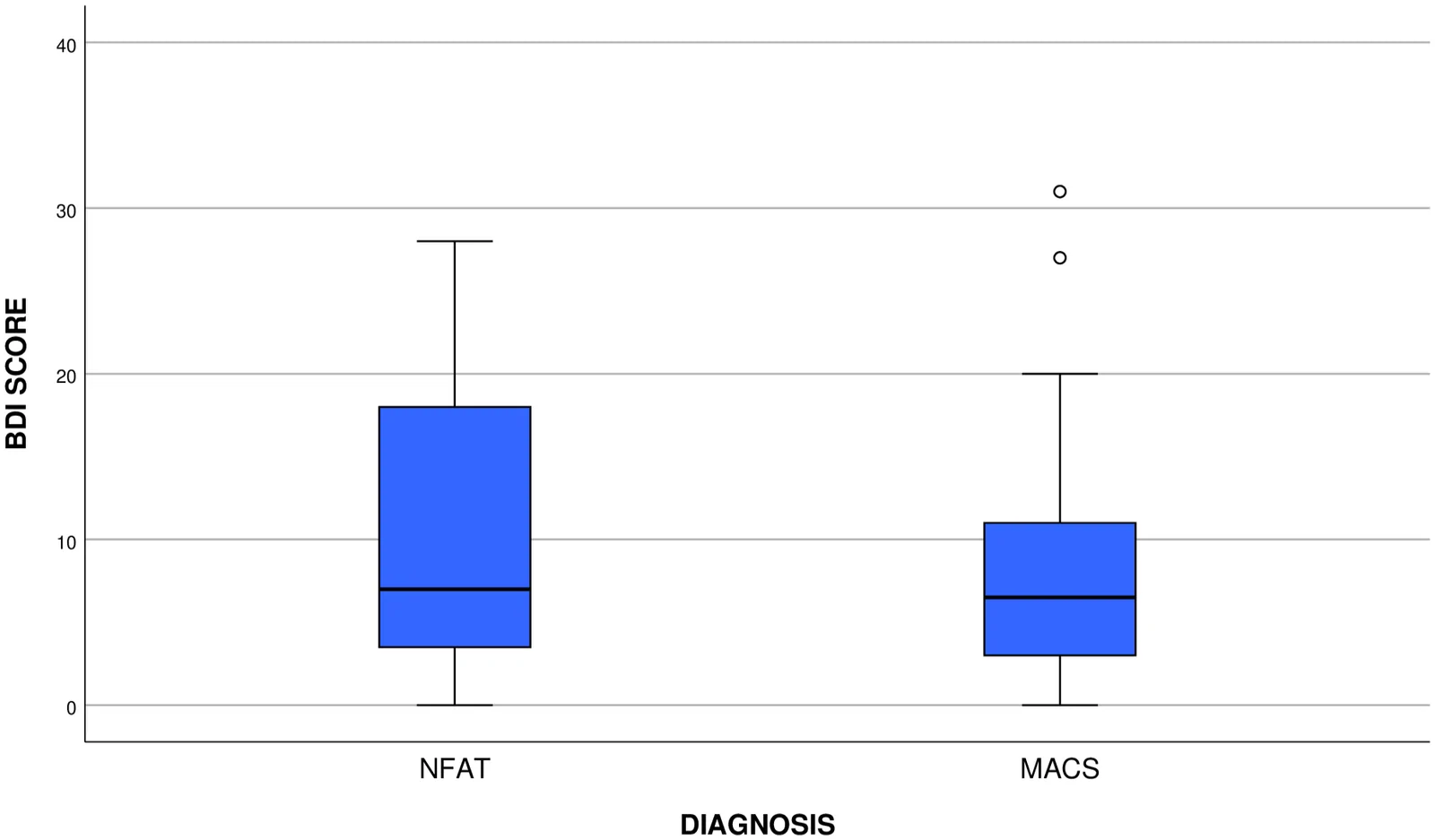
Differences in BDI scores between the NFAT and MACS groups, box plot. The box represents the interquartile range (IQR) with the median indicated by the central line; whiskers extend to 1.5 × IQR. Circles represent the outliers. NFAT, non-functional adrenal tumors; MACS, mild autonomous cortisol secretion; BDI, Beck Depression Inventory.

No significant differences were observed between the MACS and NFAT groups in terms of depressive symptoms, as measured by the BDI-II (**Figure 2**).

### 3.5 MRI findings

**Figure 3:**
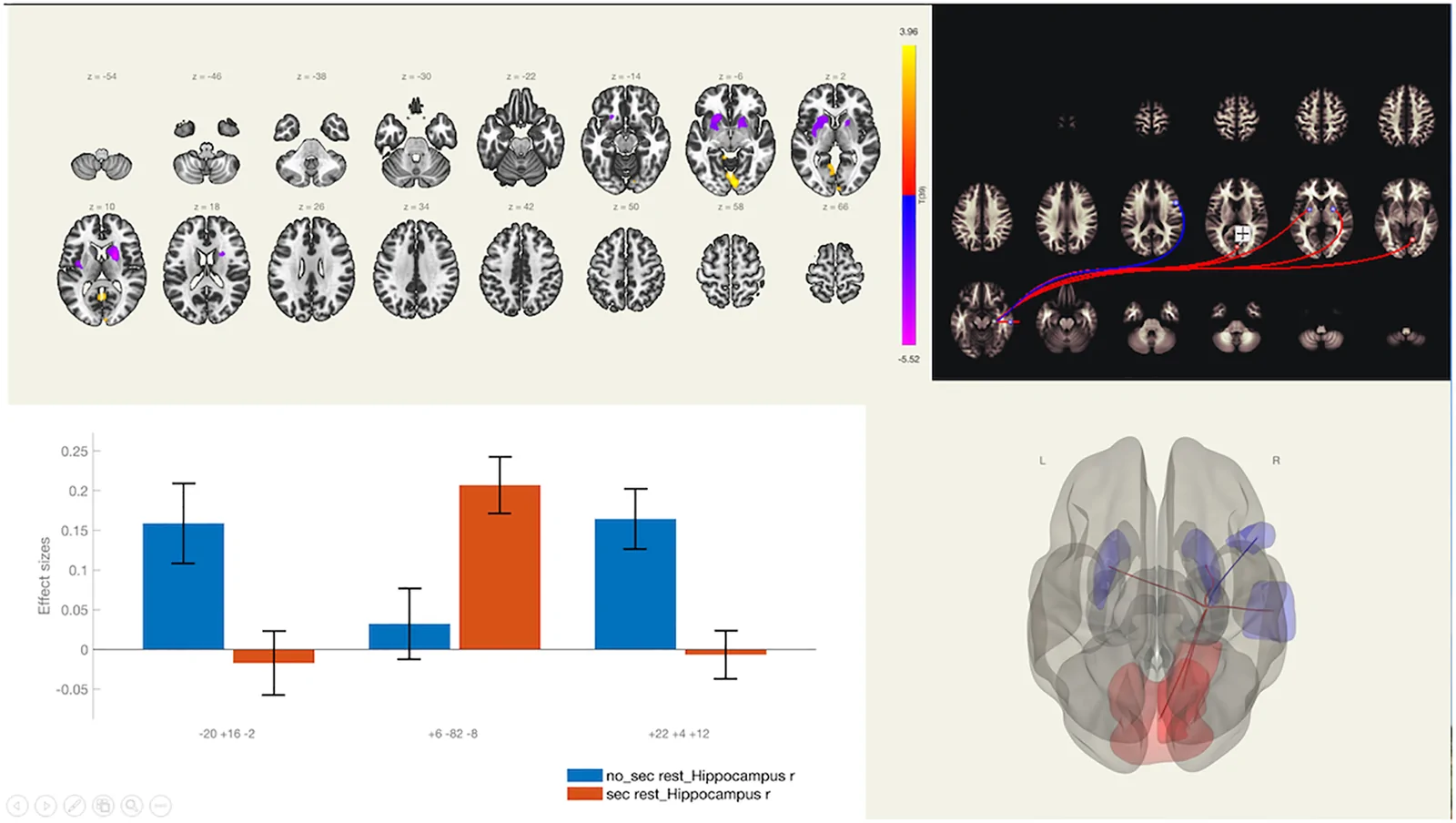
ROI-based analysis. The significant inverse correlation between the right hippocampus and the caudate nuclei and the positive correlation between the right hippocampus and the bilateral mesial occipital cortex in patients with MACS (corrected *p* < 0.05). MACS, mild autonomous cortisol secretion.

No significant differences were observed in resting-state connectivity of the major brain networks between MACS and NFAT participants. ROI-to-ROI analysis in patients with MACS revealed reduced functional connectivity between the right hippocampus and bilateral caudate nuclei, alongside increased connectivity with the bilateral mesial occipital cortex (corrected *p* < 0.05; **Figure 3**). VBM analysis revealed no significant between-group differences in gray matter volume, even after adjustment for age and sex.

## 4 Discussion

In this study, we investigated the cognitive, structural, and functional correlates of chronic cortisol exposure in patients with adrenal tumors with a multimodal approach combining neuropsychological testing, sleep and mood assessments, and structural and functional MRI analyses. Although all participants were cognitively normal, we observed subtle cognitive differences between groups. Specifically, the MACS group had slightly lower MMSE scores and worse delayed verbal recall, both negatively correlated with serum cortisol levels. These findings are consistent with prior studies suggesting that even mild hypercortisolism can selectively affect hippocampal-dependent memory processes, potentially through decreased glucose utilization, increased excitatory amino acid activity, reduced neurotrophic factors, and decreased neurogenesis (56), highlighting the vulnerability of the medial temporal lobe to prolonged glucocorticoid exposure (20, 21). Chronic cortisol exposure in patients with MACS may therefore induce early functional alterations in the hippocampal networks. This could account not only for the subtle cognitive differences observed but also for the resting-state finding of a negative correlation between the right hippocampus and the right caudate nucleus. Additional multivariable analyses showed that the association between MACS and lower MMSE scores remained significant after adjustment for sleep quality, suggesting that global cognitive performance is affected independently of sleep disturbances.

Interestingly, the MACS group demonstrated better performance in phonemic fluency and executive speed, functions typically associated with frontal lobe integrity. This unexpected finding may suggest a region-specific effect of mild hypercortisolism (56), although it could also reflect compensatory mechanisms or sample-related variability. However, the association between MACS and phonemic fluency was attenuated after accounting for sleep quality, indicating that this finding should be interpreted with caution and requires further validation.

A significant association between MACS and poor sleep quality was observed, consistent with the known effects of glucocorticoids on circadian regulation (57). One possible explanation is that compensatory mechanisms within frontal networks may help preserve cognitive efficiency despite subtle hippocampal dysfunction. These findings explain the complex relationship between cortisol, psychoneurological symptoms or signs, sleep, and mood assessment.

Disrupted sleep may represent an early clinical manifestation of cortisol dysregulation, potentially preceding cognitive decline and structural brain changes. Furthermore, the inverse correlation observed between the right hippocampus and the caudate nuclei in our functional analysis may reflect reduced connectivity, potentially impacting reward processing, sleep, and memory. The caudate nucleus is indeed involved in sleep regulation, and alterations in its structure and function have been linked to sleep duration, REM sleep, insomnia, and other sleep disorders (58). Interestingly, the same functional connectivity changes observed in the hippocampus–caudate pathway may reflect neural mechanisms potentially involved in memory and sleep regulation. As reported in Cushing’s disease, the increase of cortisol could disrupt the functional connectivity between the hippocampus and other brain networks, including the visual network (59). In MACS, the positive correlation visible between the mesial occipital cortex and the right hippocampal region could represent an early compensatory mechanism preceding structural brain alterations.

Contrary to some previous reports (19–21), we found no significant differences in depressive symptoms. One possible explanation is that mild cortisol excess may induce a state of relative hyperarousal, characterized by increased cortical excitability, potentially preserving alertness and mitigating depressive symptoms in the early stages of disease. This hypothesis is supported by prior findings linking elevated cortisol levels to cortical hyperactivation (14).

Unlike in Cushing’s disease, cortical morphological analysis revealed no significant alterations in patients with MACS. This difference may be explained by the lower glucocorticoid exposure in MACS compared with Cushing’s disease, where structures such as the hippocampus, anterior cingulate cortex, middle frontal gyrus, and amygdala are particularly susceptible to structural changes (11, 12). The lack of cortical structural alterations in MACS may explain why higher-order cognitive functions remain largely preserved, despite subtle functional changes observed in fMRI.

Several limitations should be acknowledged: the cross-sectional design limits causal inference; the small sample size may have reduced power to detect subtle effects; although groups were matched on key variables, residual confounding (e.g., comorbid sleep apnea) cannot be excluded; the absence of a healthy control group limits the ability to determine whether observed alterations are specific to MACS or reflect adrenal incidentaloma more broadly. An additional limitation concerns the absence of formal correction for multiple comparisons in the neuropsychological and correlation analyses. Given the exploratory nature of the study and the relatively small sample size, results should be interpreted cautiously. Unfortunately, our observation period was limited to only a few years, precluding a reliable evaluation of the relationship between disease duration and neuropsychological performance. Although cumulative glucocorticoid exposure may play an important role in the development of neurocognitive alterations, this hypothesis could not be investigated in the present cross-sectional study and warrants evaluation in future longitudinal studies.

Finally, the lack of longitudinal follow-up limits generalizability; longitudinal studies are needed to assess the progression of cognitive and neuroimaging alterations in MACS and their potential reversibility following adrenalectomy or medical treatment.

## 5 Conclusions

Our findings highlight the need to carefully evaluate the clinical importance and management of MACS. The observed cognitive, sleep, and functional alterations suggest that even “subclinical” cortisol excess may not be entirely benign. Future studies should explore whether these alterations progress over time or predict long-term outcomes.

## Data Availability

The raw data supporting the conclusions of this article will be made available by the authors, without undue reservation.
